# Microperforate Hymen: A Rare Case of Pregnancy and Miscarriage

**DOI:** 10.7759/cureus.67517

**Published:** 2024-08-22

**Authors:** Shruti Ganti, Tharaka Senathirajah, Rohini Govindarajan, Jayashree Srinivasan

**Affiliations:** 1 Obstetrics and Gynaecology, Saveetha Medical College and Hospitals, Saveetha Institute of Medical and Technical Sciences, Saveetha University, Chennai, IND

**Keywords:** amenorrhea, dyspareunia, vesicouretheral reflex, miscarriage, pregnancy, microperforate hymen

## Abstract

Microperforate hymen is a congenital defect where the hymenal ring covering the vaginal opening is abnormally small, leading to problems such as painful intercourse, difficulty inserting tampons, and complications during menstruation and pregnancy. The hymen, formed during fetal development, undergoes canalization before birth, with deviations in this process leading to conditions like imperforate or microperforate hymen. This case report discusses a 28-year-old primigravida diagnosed with a microperforate hymen during the management of a missed miscarriage. Following routine investigations, she underwent a hymenectomy and suction evacuation, which alleviated the immediate issue. Despite an uneventful post-operative period, the patient experienced dyspareunia three months later, necessitating further treatment with serial dilators. A microperforate hymen can cause significant reproductive health issues, including menstrual irregularities, recurrent infections, and infertility. Timely diagnosis and appropriate surgical intervention are crucial in managing this rare condition, improving patient outcomes and quality of life.

## Introduction

Microperforate hymen is a congenital defect wherein there is an obstruction to the vaginal opening, as the usual opening within the hymenal ring is smaller. This condition can lead to a range of problems, including painful intercourse, difficulty inserting tampons, and, in some cases, difficulty with menstruation and even pregnancy [1]. During embryonic development, most of the genital tract develops from the Wolffian (mesonephric) ducts and Mullerian (paramesonephric) ducts in males and females, respectively [2]. The hymen develops at the junction of the sinovaginal bulb and the urogenital sinus at the distal end of the vaginal canal, which forms a thin membrane. This development seems to occur during the 22nd week of pregnancy.

During the development of a female fetus, the hymen, which is a thin membrane at the opening of the vagina, typically forms with an opening. This process is known as canalization. If this process doesn’t occur properly, it can lead to conditions like a microperforate hymen, where the opening is unusually small, or an imperforate hymen, due to the absence of epithelial cell degradation. In cases of a microperforate hymen, partial absence is observed. The hymen undergoes canalization before birth, and the shape and diameter of the orifice can vary. Initially, conditions such as imperforate hymen or vaginal septum were considered; however, the presence of a small hymenal opening with menstrual blood flow ruled out these possibilities and confirmed the diagnosis of a microperforate hymen. The hymen is the link between the vagina and the external environment, helping cervicovaginal secretions and menstrual blood to pass through after menarche. An imperforate hymen is usually associated with other anomalies, like duplex kidney, hydronephrosis, and vesicoureteral reflux, which are reported to occur in 4.2 per 10,000 live births [3]. It is often diagnosed by the time of puberty, as it presents with cyclical pain associated with primary amenorrhea. On the contrary, the diagnosis of a microperforate hymen can be delayed, as menstruation can still occur. A microperforate hymen may be associated with recurrent vulvovaginitis and urinary tract infections. Dyspareunia and infertility are other symptoms of this condition. A high degree of suspicion is thereby needed for early diagnosis and timely intervention.

In cases where a woman with a microperforate hymen becomes pregnant, there is a high risk of complications during pregnancy and childbirth. Though the conception rates are comparatively low, pregnancy has been reported in women with a microperforate hymen. However, it may be associated with labor complications, such as prolonged labor due to soft tissue obstruction or increased operative deliveries. Despite its rarity, a microperforate hymen can have significant impacts on a woman's reproductive health and quality of life. Early diagnosis and appropriate management can help prevent complications and improve outcomes for affected women. Here, we discuss a case of a primigravida with a microperforate hymen and miscarriage [4].

## Case presentation

A 28-year-old primigravida, at 10 weeks and one day of gestational age, conceived spontaneously after one and a half years of marriage. Pregnancy was confirmed by a urine pregnancy test at 45 days of amenorrhea. She presented with complaints of per-vaginal spotting. There was no history of lower abdominal pain, fever, foul-smelling discharge, or burning micturition. She had attained menarche at 13 years of age, and her cycles were regular, with no clots nor dysmenorrhea. On further probing, she revealed a history of dyspareunia. She was also a known diabetic and was on oral hypoglycemic medications for the same. She had been on regular folate tablets as well. Upon probing the past history, she revealed that she was on tablet oxcarbazepine 300 mg once daily for a neuropsychiatric illness.

On examination, her general condition was fair, and she did not have any pallor or pedal edema. Her systemic examination was normal; her thyroid and breast were clinically normal. Per abdomen examination was normal. Speculum could not be inserted for a per speculum examination. On per vaginal examination, the hymen ring presented with a small opening, and minimal altered color blood was noted. Upon routine investigations on admission, she was diagnosed as hypothyroid and was started on Thyronorm 25 micrograms once daily. Ultrasound imaging showed a single intrauterine pregnancy with absent fetal cardiac activity. Other investigations were found to be normal. She was diagnosed with a microperforate hymen and a missed miscarriage. She was planned for concurrent hymenectomy with suction and evacuation after obtaining anesthesia fitness.

**Figure 1:**
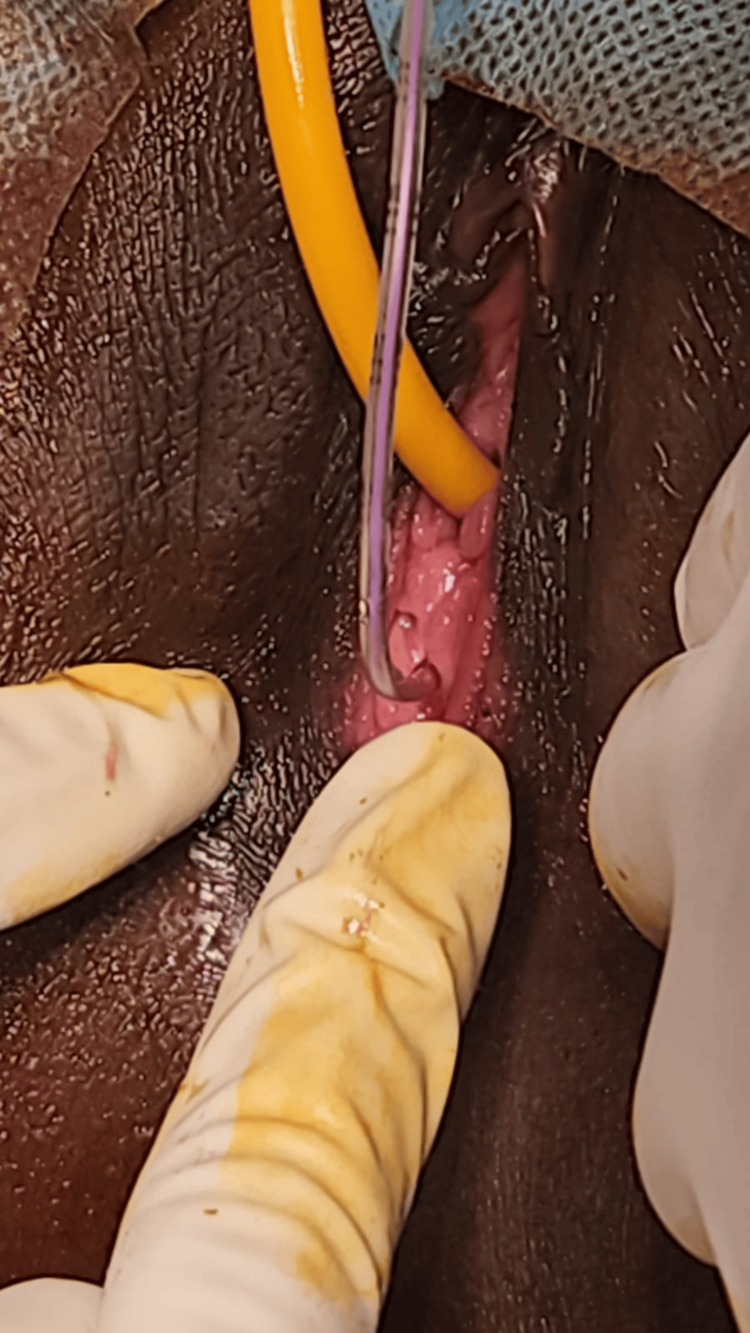
The pinhole opening in the hymen, through which an infant feeding tube was passed, illustrates the restricted vaginal opening characteristic of a microperforate hymen

**Figure 2:**
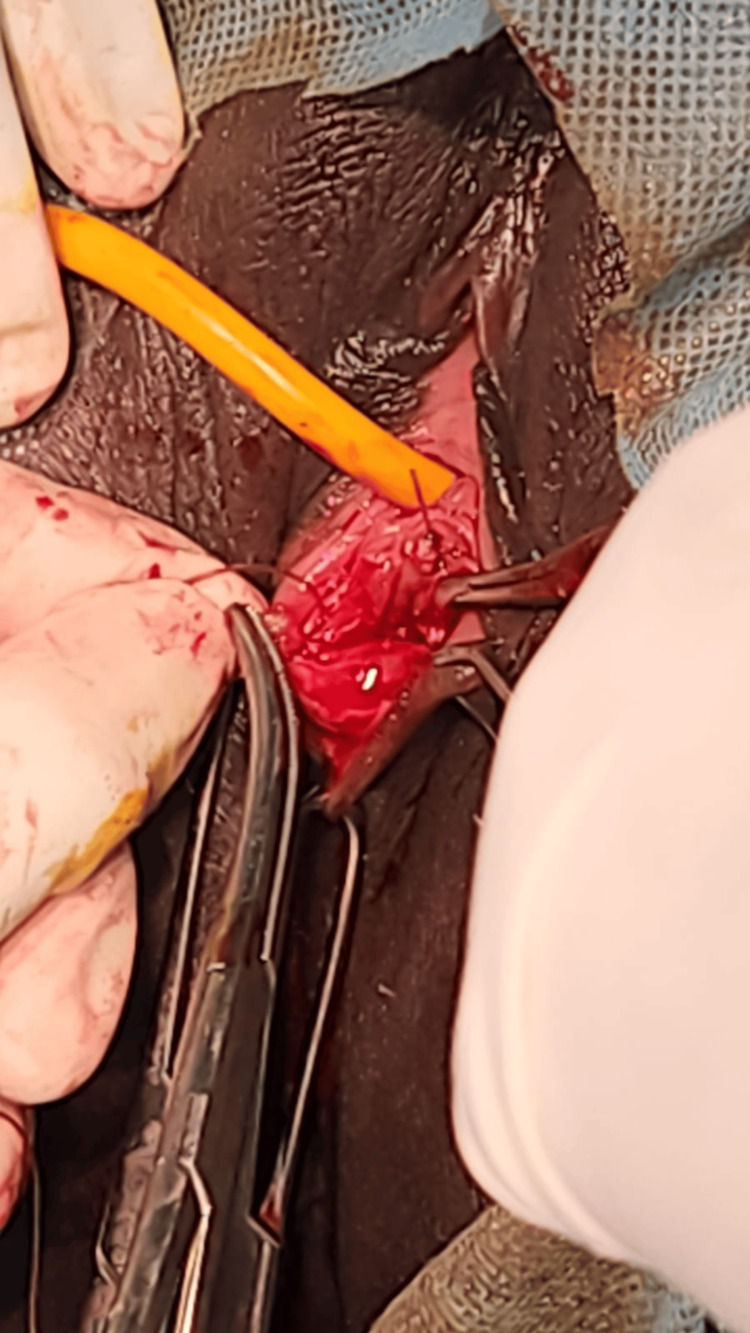
Cruciate incision and edges sutured

During the procedure, after bladder catheterization, an infant feeding tube was introduced through the microperforation in the hymen, as shown in Figure 1. As illustrated in Figure 2, the cruciate incision technique was employed to enlarge the hymenal opening, facilitating subsequent suction evacuation. The edges of the hymen were sutured to the labia minora all around using a 2-0 chromic catgut. A speculum examination was done; it was able to hold in place, and the cervix was found high up, with minimal serosanguinous discharge in the vaginal canal. This was followed by suction evacuation with a no. 6 Karmann’s cannula, and the products of conception were removed.

**Figure 3:**
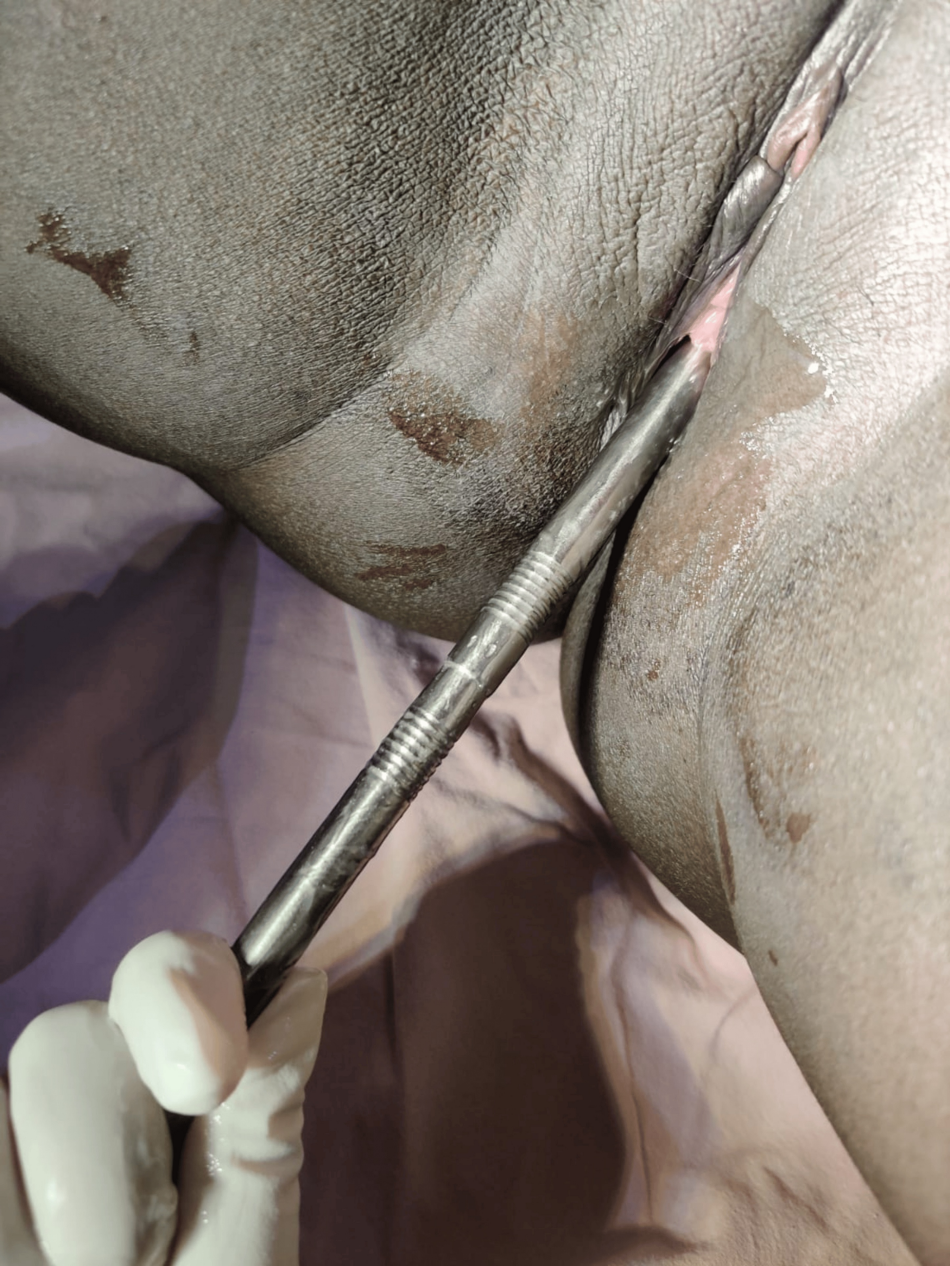
The cruciate incision technique was employed to enlarge the hymenal opening, facilitating subsequent suction evacuation

The post-operative period was uneventful, and healing was good. No undue bleeding or infections were noted. The patient was advised to take all her medications regularly and was discharged with advice to follow up in two weeks to check for healing. The patient failed to follow up in the given time but presented after three months with complaints of dyspareunia. On examination, it was deduced that only the little finger could be passed through the opening. Hence, serial dilatation with mechanical dilators was done, following which the opening increased in size, as shown in Figure 3. She was educated on the use of serial dilators at home and was discharged.

## Discussion

Microperforate hymen incidence ranges from 1 in 1,000 to 1 in 2,000. It occurs when the hymen, which is a thin membrane that partially covers the vaginal opening, has only a small opening or multiple tiny perforations. Unlike a normal hymen, which has a central opening, the microperforate hymen has a thin tissue layer covering most of the vaginal opening, making it difficult for menstrual blood to flow out, for sexual intercourse, and for childbirth to occur.

The exact cause of microperforate hymen is unknown, but it is believed to be a congenital condition that develops during fetal development. It is not associated with any underlying medical conditions or genetic abnormalities. In most cases, microperforate hymen is asymptomatic, and women may not even be aware that they have this condition until puberty. As menstruation begins, menstrual blood may collect in the vagina and cause abdominal pain, cramps, and swelling of the lower abdomen. Some women may also experience back pain, headaches, and nausea during their menstrual cycles.

Other symptoms of microperforate hymen may include difficulty inserting tampons, pain during sexual intercourse, and recurrent urinary tract infections. In severe cases, the accumulation of menstrual blood can lead to a vaginal or uterine infection, causing fever, chills, and general malaise. Microperforate hymen is diagnosed through vaginal examination, which may reveal abnormalities such as the thickness of the hymen and the size of the vaginal opening. In some cases, an ultrasound or MRI may be necessary to confirm the diagnosis [5].

Spontaneous conception has been reported in the literature in women with microperforate hymen, as the pinhole opening sometimes allows the passage of seminal fluid into the vaginal canal even without complete penetration. As the pregnancy progresses, the woman may experience difficulty with normal vaginal delivery, as the passage is obstructed. There have also been reports of second-trimester miscarriages.

Similar to our case, Sharma et al. [6] reported a medical termination of a second-trimester anomalous fetus, who presented with abdominal pain and bleeding per vaginum after ingestion of MTP pills, in which microperforate hymen was an incidental finding on routine examination. This underscores the importance of clinical case examination and highlights that a high degree of suspicion is warranted. Microperforate hymen is usually diagnosed late, as menstrual irregularities may be noted only later; however, recurrent urinary tract infections can be an important adjunctive diagnosis. The underlying cause has been postulated to be due to urinary retention caused by a partial obstruction of the urethra by entrapped vaginal secretions (e.g., hydrocolpos or mucocolpos). Another cause is the entrapped bacteria in the vaginal canal ascending upwards.

Tardieu and Appelbaum [7] have reported a case of pyocolpos and microperforate hymen in a three-year-old child. This explains that untreated microperforate hymen can be a cause of ascending pelvic infection and recurrent vulvovaginitis. It is interesting to note that, over the past years, very few cases of microperforate hymen have been documented [8-10]. Microperforate hymen, in itself, is not a cause of miscarriage, but if miscarriage cases present with disproportionate abdominal pain and absent bleeding, suspicion should be raised for an incidental finding of a microperforate hymen. When diagnosed, it becomes mandatory to concurrently treat the underlying congenital abnormality to prevent further complications and to help in alleviating the existing symptoms of dyspareunia and infections, and, in the case of the reproductive age group, allow for a successful pregnancy.

Hymenotomy is a simple surgical procedure that is performed under local or general anesthesia [11]. During the procedure, a small, usually cruciate incision is made in the hymen, creating a larger opening to alleviate the issues. After the procedure, the patient may experience mild discomfort, bleeding, and vaginal discharge for a few days, but most women resume normal activities within a week. As in our case, the procedure may warrant serial dilatation of the vagina with mechanical dilators after the hymenotomy to keep the vaginal opening patent until the couple resumes regular intercourse. Thus, besides the regular surgical procedures, non-surgical options like mechanical serial vaginal dilators are available [12,13].

It is essential to note that not all cases of microperforate hymen will result in miscarriage. With proper management and monitoring, many women with this condition can go on to have healthy pregnancies and childbirth. In a few cases, as the pregnancy reaches term, prolonged labor or obstructed labor with no identified fetal or obstetric cause warrants a high degree of suspicion to look for any abnormalities in the passage, and the condition may be diagnosed incidentally during labor. Elshani et al. [14] reported a case of a primigravida at 39 weeks of pregnancy who was delivered by cesarean section because the hymenal ring resulted in obstructed labor, and hymenotomy was also performed concurrently. However, early diagnosis and appropriate management are crucial to prevent complications and improve outcomes for affected women.

Microperforate hymen has also been associated with infertility, though an indirect cause. After an initial workup for the primary causes of infertility, and a complete history and examination, the patient might present with complaints of incomplete penetration, severe pain during intercourse, and abdominal pain. Guven et al. [15] and Baskaran and Sharma [2] have reported cases of women experiencing difficulties in conception, with a normal hormonal profile, no menstrual irregularities, no adverse medical conditions, and a normal uterus and ovaries on ultrasound. Further evaluation revealed a microperforate hymen, which might be preventing the easy passage of seminal fluid into the vaginal canal. In some cases, an associated vaginal septum might be encountered, but in most cases of microperforate hymen, no additional findings are seen. In contrast, cases of imperforate hymen can be associated with renal anomalies, such as a duplex kidney.

Microperforate hymen is an entity not very well explained in the literature, as the microperforations can be as small as a pinhole, might have small cribriform multiple openings, and are not big enough to cause any menstrual irregularities. If left untreated, microperforate hymen can lead to serious complications, including pyometra, urinary tract infections, and sepsis. The accumulation of menstrual blood can cause inflammation, which can lead to scarring, vaginal stenosis, adhesions, endometriosis, and infertility. In some cases, microperforate hymen can lead to complications during childbirth, such as obstructed labor, uterine rupture, and postpartum hemorrhage.

## Conclusions

Microperforate hymen, characterized by an insufficiently perforated hymenal membrane, can lead to issues such as menstrual obstruction and discomfort. Understanding the subtle presentations of this condition allows for accurate diagnosis, often involving a detailed patient history and careful physical examination. Effective management typically involves a surgical intervention to correct the hymenal opening, which can alleviate symptoms and restore normal menstrual function. Post-operative care and follow-up are essential to monitor healing and address any residual concerns. By recognizing the signs of a microperforate hymen early and employing a tailored treatment approach, healthcare providers can significantly enhance patient well-being and prevent long-term complications associated with this condition. Long-term follow-up is recommended to monitor for the recurrence of symptoms, such as dyspareunia, and to ensure that the patient’s reproductive health is maintained. The successful management of this case aligns with outcomes reported in similar cases, underscoring the effectiveness of early surgical intervention in patients with microperforate hymen.
